# *SWEET* Gene Family in *Medicago truncatula*: Genome-Wide Identification, Expression and Substrate Specificity Analysis

**DOI:** 10.3390/plants8090338

**Published:** 2019-09-09

**Authors:** Bin Hu, Hao Wu, Weifeng Huang, Jianbo Song, Yong Zhou, Yongjun Lin

**Affiliations:** 1 National Key Laboratory of Crop Genetic Improvement and National Centre of Plant Gene Research, Huazhong Agricultural University, Wuhan 430070, China (B.H.) (W.H.); 2 Henry Fok Collge of Life Sciences, Shaoguan University, Shaoguan 512005, China; 3 College of Bioscience and Bioengineering, Jiangxi Agricultural University, Nanchang 330045, China; 4 Key Laboratory of Crop Physiology, Ecology and Genetic Breeding, Ministry of Education, Jiangxi Agricultural University, Nanchang 330045, China

**Keywords:** *Medicago truncatula*, SWEET, sugar transport, evolution, expression analysis, abiotic stress

## Abstract

SWEET (Sugars Will Eventually be Exported Transporter) proteins mediate the translocation of sugars across cell membranes and play crucial roles in plant growth and development as well as stress responses. In this study, a total of 25 *SWEET* genes were identified from the *Medicago truncatula* genome and were divided into four clades based on the phylogenetic analysis. The *MtSWEET* genes are distributed unevenly on the *M. truncatula* chromosomes, and eight and 12 *MtSWEET* genes are segmentally and tandemly duplicated, respectively. Most *MtSWEET* genes contain five introns and encode proteins with seven transmembrane helices (TMHs). Besides, nearly all MtSWEET proteins have relatively conserved membrane domains, and contain conserved active sites. Analysis of microarray data showed that some *MtSWEET* genes are specifically expressed in disparate developmental stages or tissues, such as flowers, developing seeds and nodules. RNA-seq and qRT-PCR expression analysis indicated that many *MtSWEET* genes are responsive to various abiotic stresses such as cold, drought, and salt treatments. Functional analysis of six selected MtSWEETs in yeast revealed that they possess diverse transport activities for sucrose, fructose, glucose, galactose, and mannose. These results provide new insights into the characteristics of the *MtSWEET* genes, which lay a solid foundation for further investigating their functional roles in the developmental processes and stress responses of *M. truncatula*.

## 1. Introduction

The SWEET (sugars will eventually be exported transporter) family, a novel family of sugar transporters, can mediate the diffusion of sugars across intracellular or plasma membranes and exhibits low sugar affinity [1,2]. The transport activities of SWEET proteins for both cellular uptake and efflux of various mono- and di-saccharides are dependent neither on proton gradient nor on pH, suggesting that they are energy-independent uniporters [3,4]. In addition, SWEETs could be classified into four clades (I–IV) based on their phylogenetic relationships, and members in different clades exhibit differences in subcellular localizations and substrates [1,2,5,6].

SWEETs are widely distributed in prokaryotes, plants and animals, and most of them comprise seven transmembrane helices (7-TMHs) harboring two conserved MtN3/saliva domains (PF03083), and each domain is a triple helix bundle (THB) formed by the three TM helices (3-TMHs) [7]. The eukaryotic SWEETs consist of two THBs and the remaining TMH serves as a linker in the middle of them, forming a “3-1-3” structure [1,4]. The prokaryotic SWEET proteins (designated as SemiSWEETs) contain a single THB, and the eukaryotic SWEETs might have evolved from SemiSWEETs by gene duplication and the fusion of THBs via an inversion linker helix [8,9,10]. In addition, some bacterial SWEETs with six or seven TMHs were also found, and 14-TMH SWEETs and 15-TMH extraSWEETs were identified from *Vitis vinifera* and *Oryza punctate*, respectively [11,12]. The two extraSWEETs have a duplication of 7-TMH within the genes, which is similar to the duplication of the SemiSWEETs that produce SWEETs. Moreover, three SWEETs from oomycetes contain 18, 23, and 25 TMHs, respectively, and are named as superSWEETs, with each which carrying 5–8 repeats of a SemiSWEET with 3-TMH [12,13]. These findings reveal that duplication and fusion play vital roles in the evolution of the SWEET proteins.

Many *SWEET* genes have been well studied in plants, and are considered to play key roles in the regulation of plant growth and development such as phloem loading, nectar secretion, pollen development, and seed filling. For example, *AtSWEET8/RPG1* (*ruptured pollen grain1*) encodes a glucose transporter that functions in primexine deposition and shows redundant function with *AtSWEET13/RPG2* for pollen wall pattern formation at late reproductive stage [14,15]. Maize ZmSWEET4c and its rice ortholog OsSWEET4 are associated with hexose transport across the basal endosperm transfer layer during seed filling [16]. AtSWEET9 is a nectary-specific transporter and was shown to function in the secretion of sucrose for nectar production [17]. In addition, SWEETs have been documented to play important roles in the response to abiotic stresses. For example, the *atsweet11atsweet12* double mutant, which was defective in the development of vascular bundles, displayed deficient phloem loading and retarded growth, but exhibited higher freezing tolerance due to the high accumulation of sugars in the leaves [2,18]. *SAG29/AtSWEET15* was found to be related to cell viability under high salinity and other osmotic stress conditions [19]. Both AtSWEET16 and AtSWEET17 act as fructose uniporters across the tonoplast membrane and determine the fructose content in the tonoplast of leaves and roots, and overexpression of *AtSWEET16* remarkably increased the tolerance of *Arabidopsis* to freezing stress [20,21,22]. Moreover, SWEETs also play important roles in plant-microbe interactions, possibly promoting the sugar supply for pathogen growth. For example, AtSWEET2, a vacuolar glucose transporter, can limit carbon secretion from roots, thus contributing to resistance against *Pythium* [23]. *Xanthomonas* transcription activator-like (TAL) effectors were shown to target *Xa13/Os8N3/OsSWEET11* [24,25], *Xa17/Os11N3/OsSWEET14* [26], and *Xa25/OsSWEET13* [27] in rice, and *MeSWEET10a* in cassava [28], to promote the sugar supply in infected cells. *IbSWEET10* encodes a sugar transporter protein involved in sucrose transport, and contributes to the resistance of sweet potato to *Fusarium oxysporum* [29]. These diverse functions of SWEET proteins demonstrate that they play crucial roles in various physiological processes and responses to abiotic/biotic stresses of plants.

In previous reports, the *SWEET* gene family has been characterized in various plant species, such as *Arabidopsis* [1], rice [30], sweet orange [31], soybean [11], tomato [32], potato [33], sorghum [34], cucumber [5,35], rubber tree (*Hevea brasiliensis*) [36], pineapple (*Ananas comosus*) [37], apple [38], tea plant [39], Chinese cabbage (*Brassica rapa*) [40,41], wheat [13,42], and cabbage (*B. oleracea*) [43]. However, the *SWEET* gene family in *Medicago truncatula* is still poorly understood, and the biological functions of MtSWEETs remain largely unknown. In this study, we conducted a comprehensive genome-wide analysis of the *M. truncatula* SWEET family members, including the phylogenetic relationships, chromosomal distributions, gene structures, expression patterns in different developmental stages/tissues as well as in response to cold, drought and salt stresses. In addition, we also analyzed the substrate specificity of these MtSWEET proteins to characterize their specific functions in *M. truncatula*. The results will provide a solid foundation for further characterizing the functions of MtSWEET proteins in the regulation of plant development and stress responses.

## 2. Materials and Methods

### 2.1. Identification of SWEET Genes in M. truncatula

The *M. truncatula* Mt4.0v1 protein sequences were downloaded from Phytozome 12 (https://phytozome.jgi.doe.gov/). The Hidden Markov Model (HMM) profiles of the SWEET domain (PF03083) were downloaded from the Pfam database (http://pfam.xfam.org/) and used to search the SWEET proteins in the *M. truncatula* proteome with HMMER software. In addition, the protein sequences of 17 AtSWEETs and 21 OsSWEETs were downloaded from TAIR (http://www.arabidopsis.org/) and TIGR (http://rice.plantbiology.msu.edu/), respectively, and used as queries to search against the *M. truncatula* proteome. All resulting non-redundant protein sequences were checked for the presence of the entire SWEET domain by SMART (http://smart.embl-heidelberg.de/) and InterProScan (http://www.ebi.ac.uk/interpro/). The proteins with very short amino acid sequences (<150 aa) were excluded. The ProtParam tool (http://web.expasy.org/protparam) was used to analyze the sequence length, molecular weight and theoretical isoelectric point (pI) values of each MtSWEET protein. The distributions of TM helices were predicted using the TMHMM Server v. 2.0 (http://www.cbs.dtu.dk/services/TMHMM).

### 2.2. Chromosomal Locations, Gene Duplication, Phylogenetic and Gene Structure Analysis

The information of locations for each *MtSWEET* gene was obtained in *M. truncatula* genome (version Mt4.0v1) from Phytozome 12 and mapped with the GenomePixelizer software. The duplicated genes were identified by MCScanX according to the previously described criteria [44,45]. Multiple sequence alignment was carried out considering the full-length SWEET protein sequences from *Arabidopsis* [1], sorghum [34], cucumber [35], and *M. truncatula* using the Clustal Omega program [46]. Subsequently, an unrooted neighbor-joining (NJ) tree was constructed by MEGA 7.0 with 1000 bootstrap replicates [47]. Gene Structure Display Server (GSDS) (http://gsds.cbi.pku.edu.cn/) was employed to generate a schematic diagram of the gene structure of *MtSWEET* genes by comparing the coding sequence (CDS) with their corresponding genomic DNA (gDNA) sequences.

### 2.3. In Silico Expression Profile Analysis of MtSWEET Genes

To study the developmental expression profile of the *MtSWEET* genes, genome-wide microarray data from *M. truncatula* in different tissues at various developmental stages were retrieved from *M. truncatula* Gene Expression Atlas (MtGEA, https://mtgea.noble.org/v3/), and the transcript data of the *MtSWEET* genes were analyzed as previously described [48]. The normalized expression data were used to generate heatmap using the TBtools software [49]. To investigate the expression profiles of the *MtSWEET* genes in response to abiotic stresses, genome-wide transcriptome sequencing (RNA-seq) data from *M. truncatula* under different stresses (including cold, drought, and salt) were extracted according to a previous study [50]. The raw data of this entire microarray experiment have been deposited in NCBI GEO (GSE136739). RNA-seq reads were aligned to the *M. truncatula* Mt4.0v1 genome sequences using Tophat 2.1.0 [51], and read counts for *MtSWEET* genes were normalized as fragments per kilobase of transcript per million mapped reads (FPKM) values using StringTie (v2.0) [52,53], representing the expression abundance of each *MtSWEET* gene. The expression data were employed to create the heatmaps and illustrated using the ggplot2 package in R software. Compared with the control (0 h for every treatment), differentially expressed genes (DEGs) were identified for at least one of the time points with a cutoff of fold change ≥2 or ≤0.5.

### 2.4. Plant Materials and Treatments

*M. truncatula* (cv. Jemalong A17) plants were grown on half-strength Hoagland solution, and four-week-old seedlings were subjected to cold, drought and salt stresses as described previously [50]. For drought, cold, and salt treatments, the seedlings were transferred to dry Whatman 3 MM paper in a sterile Petri at the constant temperature of 4 °C, or Hoagland solutions containing 300 mM NaCl, respectively. All tests were performed in triplicate, and the seedlings were harvested after each treatment, quickly placed in liquid nitrogen, and stored at −80 °C until use.

### 2.5. RNA Isolation and Quantitative Real-Time PCR (qRT-PCR)

Total RNA was isolated with the TransZol Reagent (TransGen, China), and cDNA was synthesized by using TransScript All-in-One First-Strand cDNA Synthesis SuperMix for qPCR (One-Step gDNA Removal) (TransGen Biotech, Beijing, China) based on the manufacturer’s instructions. The qRT-PCR reactions were performed in 20 μL reactions using 1 μL of cDNA, with the FastStar Universal SYBR Green Master (ROX) (Roche Diagnostics, Indianapolis, IN, USA), on the ABI 7500 system (Thermo Fisher Scientific, Waltham, MA, USA). The qRT-PCR conditions were as described previously [54]. Three independent biological replicates were conducted. The *M. truncatula ACTIN* gene (Medtr3g095530) was used as a standard control, and the 2^−ΔΔCT^ method was used to calculate the relative levels of gene expression. The expression levels in the control plants without treatment (0 h) were normalized to 1. Data were statistically analyzed using Duncan’s test with SPSS19 and different letters indicate statistically significant differences (*p* < 0.05). The primer sequences used in this study are listed in Table S1. 

### 2.6. Substrate Specificity Analysis of MtSWEET Proteins in Yeast

The CDSs of *MtSWEET3c*, *MtSWEET5b*, *MtSWEET7*, *MtSWEET9b*, *MtSWEET15b*, and *MtSWEET16* were amplified using specific primers and then sub-cloned into the yeast expression vector pDR196. The resulting constructs and the empty pDR196 vector (as negative control) were used to transform *Saccharomyces cerevisiae* strains EBY.VW4000 and SUSY7/ura3, and the hexose and sucrose uptake assays were performed as described previously [5]. For EBY.VW4000 cells, serial dilutions (10-, 100- and 1000-fold) were plated on solid SD media supplemented with 2% concentrations of maltose (as the control), or fructose, glucose, galactose, and mannose. For SUSY7/ura3, cells were serially diluted 10-fold (10-, 100- and 1000-fold) and spotted on solid SD media supplemented with either 2% glucose (as the control), or 2% sucrose. The transformant-containing plates were photographed after incubation at 30 °C for 3–5 d. The primer sequences used are listed in Table S1.

## 3. Results

### 3.1. Genome-Wide Identification and Phylogenetic Analysis of SWEET Family Genes in M. truncatula

A total of 25 SWEET members were identified from the *M. truncatula* genome, which were named based on their homologs in *Arabidopsis* and earlier work [33]. As shown in Table 1, the predicted MtSWEET proteins ranged from 206 (MtSWEET11) to 681 (MtSWEET2b) amino acids in length, with relative molecular weights ranging from 23.53 kDa (MtSWEET11) to 73.63 kDa (MtSWEET2b), and theoretical pIs from 5.88 (MtSWEET5a) to 9.80 (MtSWEET12) (Table 1). 

**Figure 1:**
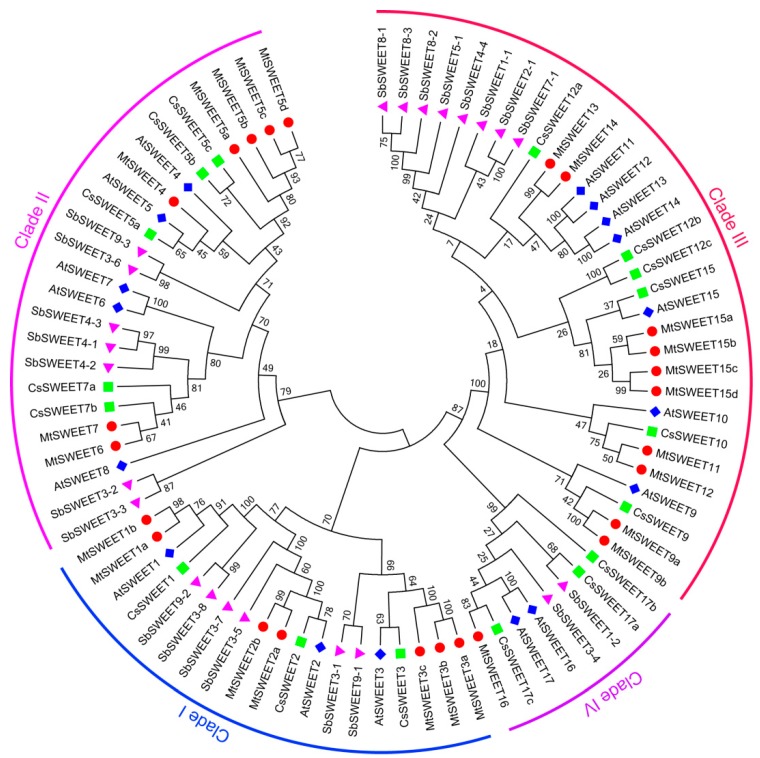
Phylogenetic relationships of the *SWEET* family genes in *Arabidopsis*, sorghum, cucumber, and *M. truncatula*. The sequences of the 82 SWEET proteins from the above four plant species were aligned by Clustal Omega, and the phylogenetic tree was constructed by the MEGA 7.0 using the NJ method with 1000 bootstrap replicates. The proteins from *Arabidopsis*, sorghum, cucumber, and *M. truncatula* are indicated with the prefixes of At, Sb, Cs, and Mt, respectively.

To explore the evolutionary relationships of *SWEET* family genes, we performed phylogenetic analysis by aligning SWEET protein sequences from *Arabidopsis*, sorghum, cucumber, and *M. truncatula* (Figure 1). In this analysis, these SWEET proteins were clearly divided into four clades (I, II, III and IV), which was in accordance with previous phylogenetic classifications of SWEETs [55]. Among them, clade III contained the largest number of MtSWEETs (10), while clade IV possessed the fewest MtSWEETs (only MtSWEET16), and clades I and II each contained seven MtSWEETs (Figure 1, Table 1).

### 3.2. Chromosomal Location and Duplication Analysis of MtSWEET Genes

**Figure 2:**
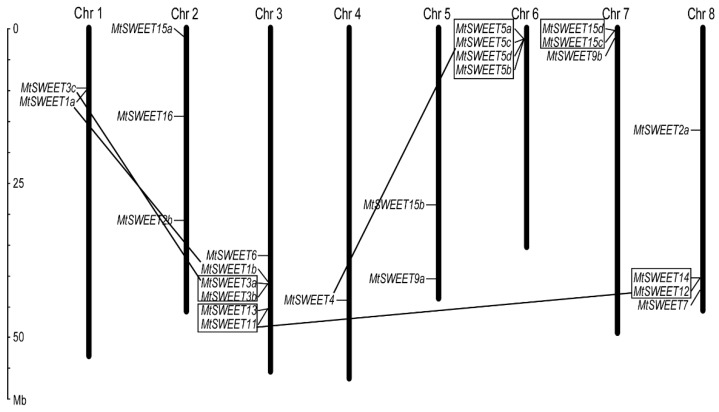
Locations and duplications of *MtSWEET* genes on *M. truncatula* chromosomes. The black lines indicate segmentally duplicated genes, and the tandemly duplicated genes are boxed. The scale is provided in megabase (Mb).

The genomic locations of the *MtSWEET* genes on *M. truncatula* chromosomes were identified. The results showed that the 25 *MtSWEET* genes were distributed on all of the eight chromosomes in *M. truncatula*. Chromosome 3 had the largest number of *MtSWEET* genes (6 genes), followed by chromosomes 6 and 8 (4 genes each), and the minimum number was found on chromosome 4 (one gene) (Figure 2). In addition, three genes were found on each of chromosomes 2 and 7, and two genes were distributed on each of chromosomes 1 and 5.

Gene duplication, including tandem and segmental duplication events, can be a crucial factor for plant genome evolution [56,57]. Based on gene duplication analysis, four segmental duplication events (*MtSWEET1a/MtSWEET1b*, *MtSWEET3a/MtSWEET3c*, *MtSWEET4/MtSWEET5c* and *MtSWEET11/MtSWEET12*) were found, and they were located on different chromosomes (Figure 2). In contrast, a total of 12 tandem duplicated genes which formed four gene clusters were found in chromosomes 3, 6, 7 and 8 (Figure 2). These results indicated that gene duplication has played a crucial role in the expansion of *MtSWEET* gene family. 

### 3.3. Analysis of Transmembrane Domains and Conserved Motifs 

The predicted results of TMHMM Server v. 2.0 suggested that most of the MtSWEET proteins contain seven TMHs, except for MtSWEET2b, MtSWEET4, and MtSWEET11 (Table 1, Figure S1). MtSWEET4 and MtSWEET11 had six TMHs, which was also observed in some SWEET members of cucumber [35] and soybean [11]. Interestingly, MtSWEET2b contained 15 TMHs (Figure S1) from the duplication of 7-TMHs, which was similar to the duplication of semiSWEET (3-TMHs) to evolve into SWEET (7-TMHs) [11,12], implying that it is an extraSWEET.

**Figure 3:**
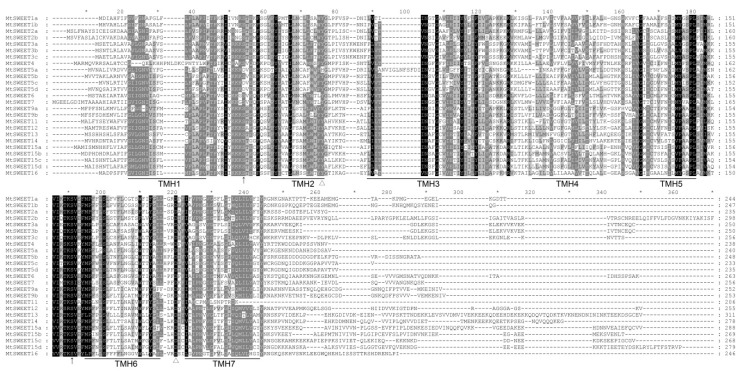
Multiple sequence alignment of MtSWEET proteins. The positions of the TMHs are underlined. The positions of the active sites of tyrosine (Y) and aspartic acid (D) are indicated by triangles. The conserved serine (S) phosphorylation sites are indicated by arrows. The sequence of MtSWEET2b was truncated.

To obtain more detailed information concerning the MtSWEET proteins, we performed multiple sequence alignments of the deduced protein sequences. As shown in Figure 3, nearly all of the MtSWEET proteins retained relatively conserved membrane domains, and contained the active sites of tyrosine (Y) and aspartic acid (D), which can form a hydrogen bond to maintain the sugar transport activity [10]. In addition, each THB contained a conserved serine (S) phosphorylation site, with the exception of MtSWEET5a, MtSWEET5c, and MtSWEET5d. The three SWEET proteins contained aspartic acid instead of serine between TMH1 and TMH2 (Figure 3). Moreover, all of the MtSWEET proteins contained the second serine phosphorylation site, which was located between TMH5 and TMH6 (Figure 3).

### 3.4. Gene Structure Analysis of MtSWEET Genes

**Figure 4:**
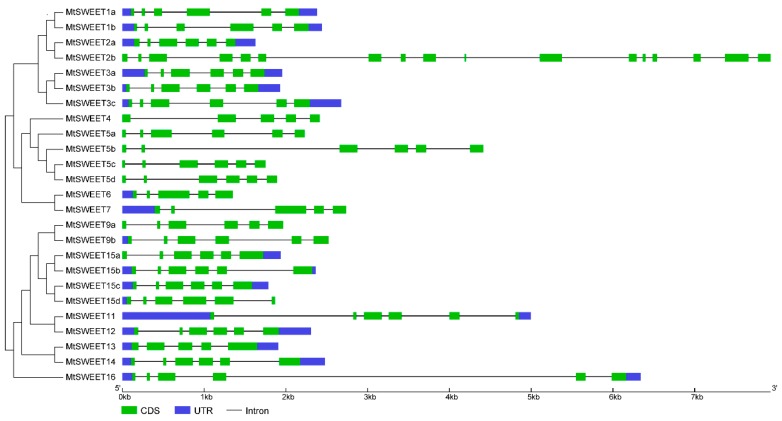
Gene structures of the *MtSWEET* genes according to their phylogenetic relationships. The blue boxes, green boxes, and black lines indicate UTRs (untranslated regions), CDSs, and introns, respectively.

To further examine the structural features of *MtSWEET* genes, the gene structures of all *MtSWEET* genes were determined by comparing the CDS and the gDNA sequences. As shown in Figure 4, the vast majority of *MtSWEET* genes (20/25) contained five introns; four *MtSWEET* genes (*MtSWEET4, MtSWEET6*, *MtSWEET7* and *MtSWEET13*) harbored four introns, while *MtSWEET2b* possessed 16 introns. In addition, genes clustered together generally exhibited similar gene structures. For example, three pairs of *MtSWEET* genes, *MtSWEET6* and *MtSWEET7*, *MtSWEET1a* and *MtSWEET1b*, *MtSWEET15a* and *MtSWEET15c*, had similar exon numbers and lengths. These results indicated that the gene structure of the *MtSWEET* family members is highly conserved.

### 3.5. Expression Patterns of Mtsweet Genes in Different Tissues

**Figure 5:**
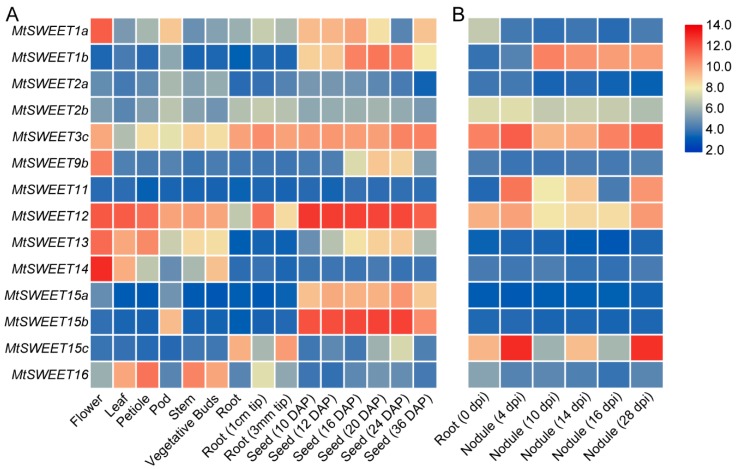
Expression analysis of the *MtSWEET* genes in different developmental tissues (**A**) and during nodule development (**B**) of *M. truncatula* using microarray data. The expression levels of the *MtSWEET* genes are shown as the log_2_-based fluorescence intensity values from the microarray data (MtGEA, https://mtgea.noble.org/v3/). DAP, days after pollination. dpi, days post inoculation.

The expression profiles of the *MtSWEET* genes in different developmental stages/tissues were examined based on the microarray data from *M. truncatula*. Eleven *MtSWEET* genes (*MtSWEET3a*, *MtSWEET3b*, *MtSWEET4*, *MtSWEET5a*, *MtSWEET5b*, *MtSWEET5c*, *MtSWEET5d*, *MtSWEET6*, *MtSWEET7*, *MtSWEET9a*, and *MtSWEET15d*) did not have corresponding probe sets in the dataset. We then the examined the expression patterns of the rest fourteen *MtSWEET* genes. As shown in Figure 5A, nearly all the *MtSWEET* genes (with the exception of *MtSWEET11*) were expressed in at least one of the tested tissues (flower, leaf, petiole, pod, stem, vegetative buds, and root). Among these genes, some exhibited relatively higher expression, such as *MtSWEET3c*, *MtSWEET12*, *MtSWEET13*, *MtSWEET14*, and *MtSWEET16*. In addition, some *MtSWEET* genes displayed obviously high expression levels in only one single tissue. For example, *MtSWEET1a*, *MtSWEET9b*, and *MtSWEET14* were preferentially expressed in flower, whereas *MtSWEET1b*, *MtSWEET15a*, and *MtSWEET15b* exhibited much higher transcript abundance in seeds than in other tissues (Figure 5A). In addition, *MtSWEET15c* also showed preferential expression in the root and seed at 24 days after pollination (DAP). 

We also analyzed the expression of these *MtSWEET* genes during nodule development (Figure 5B). Several *MtSWEET* genes were differentially expressed in response to rhizobium inoculation, such as *MtSWEET1b*, *MtSWEET2b*, *MtSWEET3c*, *MtSWEET11*, *MtSWEET12*, and *MtSWEET15c*. Among these genes, the transcriptions of *MtSWEET3c*, *MtSWEET11*, and *MtSWEET15c* were markedly enhanced at 4 or 28 days post inoculation (dpi) (Figure 5B), suggesting that they may play important roles in nodule development. Overall, our results revealed that different *MtSWEET* genes might function in diverse tissues of *M. truncatula*.

### 3.6. Expression Patterns of the MtSWEET Genes in Response to Abiotic Treatments

**Figure 6:**
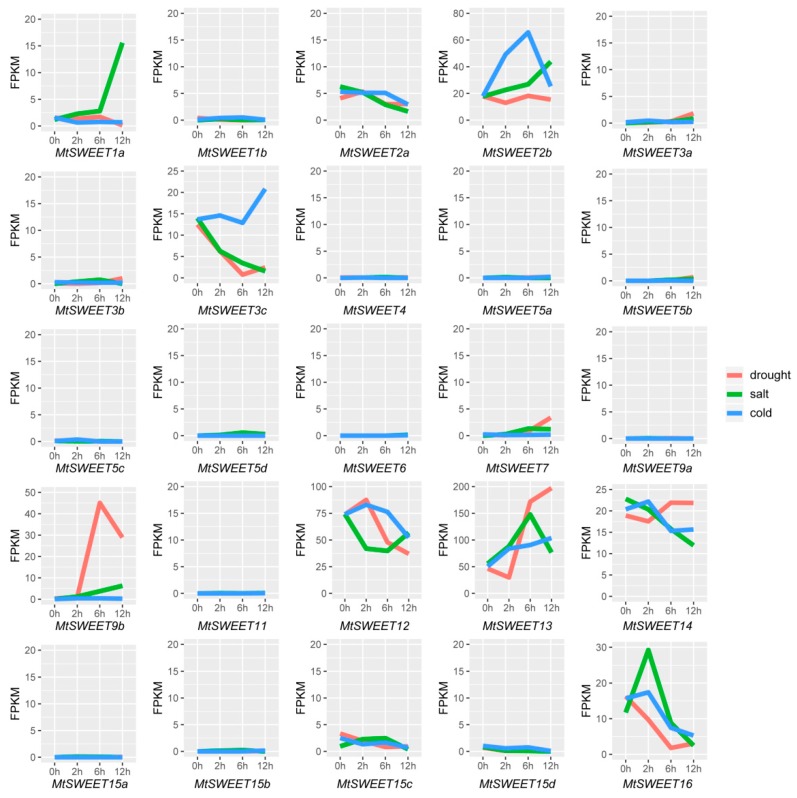
Expression patterns of *MtSWEET* genes in response to cold, drought, and salt stresses. The expression levels of the *MtSWEET* genes are shown as the FPKM values based on the RNA-seq data in our previous study [50].

To assess the potential functions of the *MtSWEET* genes in response to abiotic stress in *M. truncatula*, their expression patterns under drought, salt and cold stresses were analyzed by high-throughput sequencing data [50]. In the transcriptome analysis using RNA-seq data, *MtSWEET* genes with FPKM values lower than 1 were considered to be barely expressed. The RNA-seq data indicated that 12 *MtSWEET* genes were responsive to the three abiotic stress conditions (Figure 6; Table S2). Under cold stress, two (*MtSWEET2b* and *MtSWEET13*) and four *MtSWEET* genes (*MtSWEET1a*, *MtSWEET15c*, *MtSWEET15d*, and *MtSWEET16*) were significantly up- and down-regulated at certain time points (|log2 fold change| ≥ 1), respectively (Figure 6; Table S2). A total of eight *MtSWEET* genes showed significant changes in their expression levels under drought stress, including four up-regulated genes (*MtSWEET3a*, *MtSWEET3b*, *MtSWEET9b*, and *MtSWEET13*) and another four down-regulated genes (*MtSWEET1a*, *MtSWEET3c*, *MtSWEET15c*, and *MtSWEET16*), while the expression of *MtSWEET7* showed a decrease at 2 h but remarkable increases at 6 h and 12 h (Figure 6; Table S2). Under salt stress, five (*MtSWEET1a*, *MtSWEET2b*, *MtSWEET7*, *MtSWEET9b*, and *MtSWEET13*) and two *MtSWEET* genes (*MtSWEET2a* and *MtSWEET3c*) were significantly up-regulated and down-regulated, respectively, while the transcripts of *MtSWEET15c* and *MtSWEET16* showed an obvious increase at 2 h but a sharp decrease at 12 h (Figure 6; Table S2). These results indicated that *MtSWEET* genes play important roles in the responses to different stress treatments.

**Figure 7:**
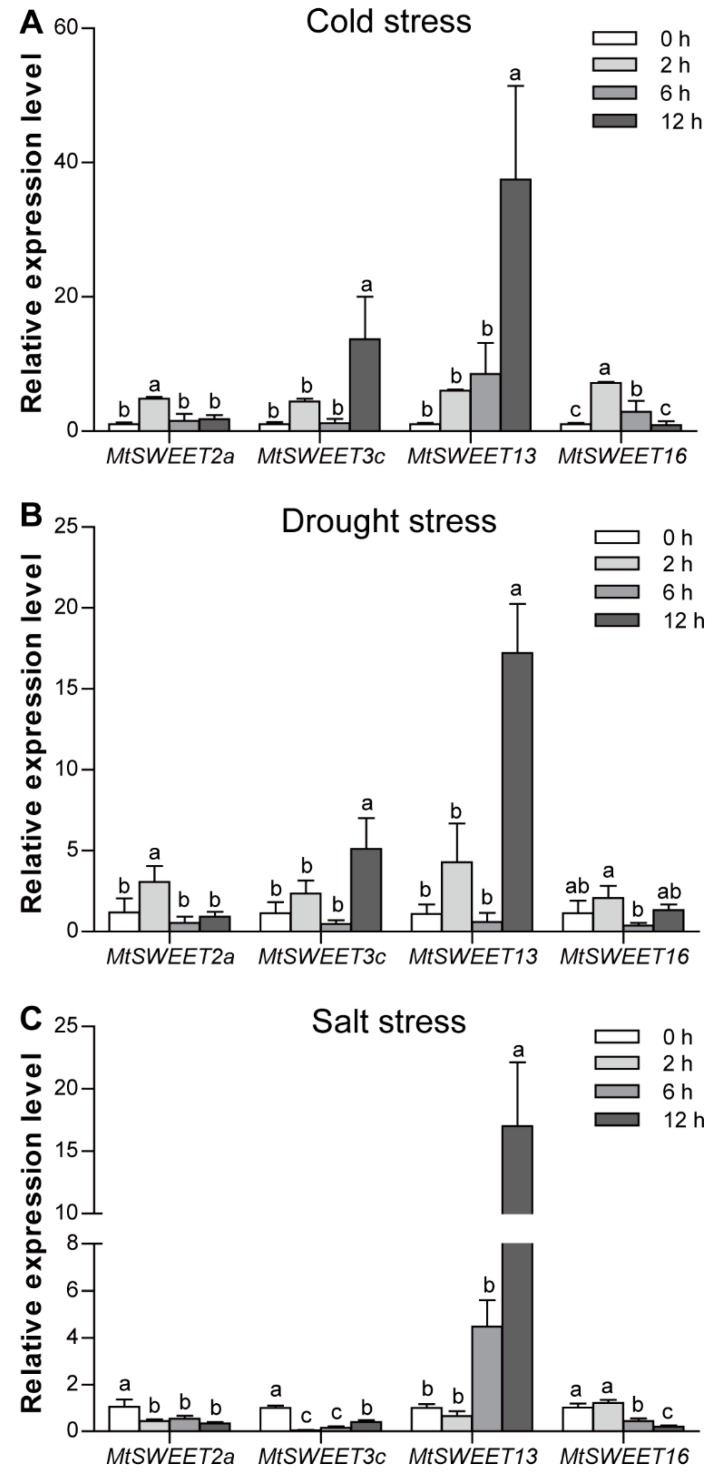
Expression levels of four selected *MtSWEET* genes in response to cold (**A**), drought (**B**), and salt (**C**) stress conditions. Expression of the genes at 0 h was set as 1, and different letters indicate statistically significant differences (Duncan’s test, *p* < 0.05).

The qRT-PCR was performed to verify the transcript levels of four selected *MtSWEET* genes in response to the above stresses. As shown in Figure 7, consistent with the results of the RNA-seq data analysis, the expression of the selected *MtSWEET* genes was significantly changed by the three stress treatments, indicating the reliability of RNA-seq data. However, there were some differences between the results of qRT-PCR and RNA-seq. For example, under drought treatment, the transcription of *MtSWEET3c* increased and peaked at 12 h in the qRT-PCR data (Figure 7B), while it was suppressed in the RNA-seq data (Figure 6; Table S2).

### 3.7. Yeast Complementation Assays of MtSWEET Proteins

**Figure 8:**
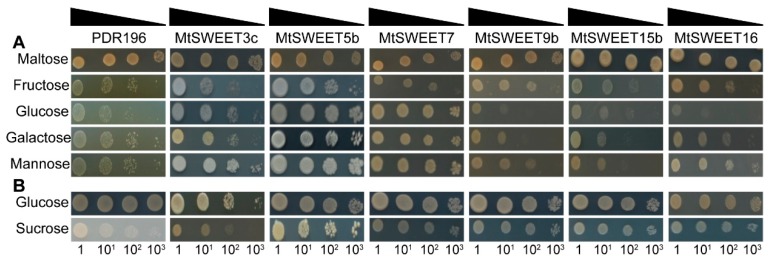
Substrate specificity analysis of six selected MtSWEET proteins in yeast mutant strains EBY.VW4000 (**A**) and SUSY7/ura3 (**B**). Cells were serially 10-fold diluted (10-, 100- and 1000-fold) and spotted on solid SD media supplemented with 2% concentration of different sugar substrates. Maltose and glucose were the sole carbon sources for the positive controls of EBY.VW4000 and SUSY7/ura3 cells, respectively.

To investigate the sugar transport abilities of MtSWEET proteins, six *MtSWEET* genes were expressed in the yeast mutants of EBY.VW4000 and SUSY7/ura3. Compared with the transformants expressing pDR196, MtSWEET5b allowed the uptake of fructose, glucose, galactose, and mannose, MtSWEET7 enabled the uptake of glucose, galactose, and mannose but not fructose, while MtSWEET3c was able to restore the growth on the medium supplemented with glucose and mannose (Figure 8A). However, MtSWEET16 only enabled mannose uptake in the yeast EBY.VW4000 mutant (Figure 8A). In addition, MtSWEET5b, MtSWEET7, MtSWEET9b, MtSWEET15b, and MtSWEET16 conferred sucrose uptake in the yeast SUSY7/ura3 mutant (Figure 8B). These results revealed that MtSWEETs might be involved in plant growth by regulating the transport of sugars.

## 4. Discussion

### 4.1. SWEET Genes in M. truncatula and Their Evolution

The *SWEET* family genes play diverse physiological and biological roles in plants. In this study, we identified 25 *SWEET* genes in the genome of *M. truncatula*, and the number was comparable to that in various plant species, such as *Arabidopsis* (17 genes) [1,2], cucumber (17 genes) [5,35], rice (21 genes) [30], sorghum (23 genes) [34], banana (25 genes) [32], tomato (29 genes) [32], cabbage (30 genes) [43], potato (35 genes) [33], and rubber tree (36 genes) [36]. Gene duplication, including tandem and segmental duplication events, can be a crucial factor that affects plant genome evolution [56,57], and contributes to the expansion of *SWEET* gene family in various plant species [12,58]. For example, two and seven pairs of tandemly duplicated genes were detected in cucumber and cotton, respectively [35,59]. In wheat, a total of 22 tandem duplication events and five segmental duplication events were identified [13]. In the present study, a total of 12 and eight *MtSWEET* genes were found to be tandemly and segmentally duplicated (Figure 2). It is noteworthy that MtSWEET2b is an extaSWEET that contains 15 TMHs (Table 1, Figure S1) derived from the duplication of 7-TMHs, and this sub-type of SWEETs has also been reported in other plants, such as grape (*V. vinifera*) [11], wild rice (*O. punctata*) [12], and cabbage (*B. oleracea*) [43]. The results revealed that the *SWEET* gene family in *M. truncatula* has also undergone expansion during the plant’s evolution.

Similar to the case in other plants [1,35,36], MtSWEETs can be classified into four clades, and the number of members in clade III is larger than that other clades (Figure 1). In addition, most *MtSWEET* genes possess six exons and five introns, which is consistent with the results in other plants, such as tomato [32], soybean [11], pear [60], *H. brasiliensis* [36], cucumber [35], and *B. rapa* [40,41], suggesting that the *SWEET* genes have been highly conserved during evolution. Moreover, the *MtSWEET* genes clustered together exhibited roughly the same gene structures in terms of the amounts of introns and lengths of exons (Figure 4), implying their similar functional roles in *M. truncatula*. However, some closely related *MtSWEET* genes displayed similar exon/intron structures but different exon/intron numbers, such as *MtSWEET4/MtSWEET5a* and *MtSWEET13/MtSWEET14* (Figure 4), suggesting the occurrence of intron/exon gain or loss events during the evolution of *SWEET* family genes in *M. truncatula*. 

### 4.2. Substrate Specificity of MtSWEET Proteins

Previous studies have suggested that the SWEETs of clades I and II prefer to transport hexose including glucose, fructose and galactose, and the SWEETs of clade III mainly transport sucrose, while those of clade IV mediate unidirectional transport of fructose [1,2,20,21,22]. In this study, yeast complementation assays were employed to examine the capability of MtSWEET proteins to transport sugars, including fructose, glucose, galactose, mannose, and sucrose. Among the detected MtSWEETs, a clade I MtSWEET member, MtSWEET3c, was found to function as a putative hexose transporter, contributing to the uptake of glucose and mannose in the yeast EBY.VW4000 mutant (Figure 8A). Another clade I MtSWEET member, MtSWEET1b, was found to function as a glucose transporter in the yeast mutant EBY.VW4000 [61]. In addition, two clade II MtSWEETs, MtSWEET5b and MtSWEET7, exhibited transport abilities for not only hexoses, but also sucrose (Figure 8). The sucrose transport activity of clade I and II SWEETs has also been reported in other plants. For example, LjSWEET3 can transport sucrose but not glucose when expressed in yeast [62]. Tea plant CsSWEET1a can also restore the yeast growth on media supplemented with sucrose [39]. A previous study has shown that a clade III member MtSWEET11 mediates the transport of sucrose but not that of glucose when expressed in HEK293T cells [63]. In this study, two other clade III members (MtSWEET9b and MtSWEET15b) were found to mainly utilize sucrose as substrate. Similarly, sweet potato IbSWEET10, cotton GhSWEET12, cucumber CsSWEET10 and CsSWEET12c enabled sucrose uptake in the yeast SUSY7/ura3 mutant but did not allow hexose uptake in yeast EBY.VW4000 mutant [5,29,64]. Furthermore, many clade III members, such as ZmSWEET13a, ZmSWEET13b, ZmSWEET13c, OsSWEET11, AtSWEET11, and AtSWEET12, have been characterized as sucrose transporters for phloem loading [2,65,66]. Members of clade IV (AtSWEET16 and AtSWEET17) were shown to mediate the transport of fructose [20,21,22], but cucumber CsSWEET17a functions in glucose uptake and CsSWEET17c functions in the uptake of glucose, galactose, and fructose in yeast [5]. In this study, MtSWEET16 was the only member in clade IV and could restore the growth of *M. truncatula* on the medium containing mannose and sucrose (Figure 8), suggesting its particular role in *M. truncatula*. Similarly, tea plant CsSWEET17 can also rescue the uptake of glucose, sucrose, fructose, galactose, and mannose when expressed in yeast [39]. Therefore, SWEETs might play distinct biological roles in *M. truncatula* through transporting different sugar molecules.

### 4.3. Functions of MtSWEET Genes in Different Tissues and in Response to Various Abiotic Stresses

*SWEET* genes have been shown to have different expression patterns to exert various functions during plant growth and development. In this study, a total of fourteen *MtSWEET* genes were found to be expressed based on the MtGEA data, and the expression of these *MtSWEET* also exhibited spatial differences. For example, several *MtSWEET* genes, including *MtSWEET1a*, *MtSWEET9b*, *MtSWEET13* and *MtSWEET14*, exhibited the highest expression levels in flowers (Figure 5A), indicating that they may participate in sugar transport for flower development of *M. truncatula*. AtSWEET9 functions as a nectary-specific sucrose transporter and plays a vital role in nectar secretion [17]. MtSWEET9b is an ortholog of AtSWEET9, and also catalyzes the transport of sucrose (Figure 8). Therefore, it can be speculated that MtSWEET9b may have a similar function to AtSWEET9. Besides the flower, three clade III members (*MtSWEET12*, *MtSWEET13*, and *MtSWEET14*) were also highly expressed in the leaf, implying that they may be involved in phloem loading and long-distance translocation of sucrose, just like their orthologs such as AtSWEET11 and AtSWEET12 [2], ZmSWEET13a, b and c [66]. In addition, *MtSWEET1b*, *MtSWEET12*, *MtSWEET15a*, and *MtSWEET15b* were highly expressed in developing seeds (Figure 5A), implying their potential roles in seed filling. ZmSWEET4c and its rice ortholog OsSWEET4 display high expression during seed development and play a key role in seed filling by improving the mobilization of hexoses into the endosperm [16]. OsSWEET11 and OsSWEET15 contribute to sucrose translocation towards the developing endosperm for seed filling [67,68]. AtSWEET11, AtSWEET12 and AtSWEET15 also display specific spatiotemporal expression patterns in developing seeds, and play a necessary role in seed filling by regulating sucrose efflux during seed development [69]. Moreover, several *MtSWEET* genes, such as *MtSWEET1b*, *MtSWEET3c*, *MtSWEET11*, *MtSWEET12*, and *MtSWEET15c*, were preferentially expressed during nodule development (Figure 5B), indicating their roles in the sugar efflux that eventually feeds the symbionts [70]. In previous studies, the expression of *MtSWEET15c/MtN3* and *MtSWEET11* was found to be restricted to the nodule [63,71], and no symbiotic defects were observed in the *mtsweet11* mutants [63], indicating that other sucrose transporters may compensate its function. Very recently, MtSWEET1b was found to function in the transport of glucose across the peri-arbuscular membrane to maintain arbuscules for a healthy mutually beneficial symbiosis [61]. Therefore, *MtSWEET1b*, *MtSWEET3c*, *MtSWEET11*, *MtSWEET12*, and *MtSWEET15c* may contribute to nodule development with seemingly redundant functions. 

Source-to-sink transport of sugars can modify the carbohydrate distribution and homeostasis that contribute to the tolerance of plants to abiotic stresses [54,72]. Many *SWEET* genes were regulated by various abiotic stresses, suggesting that they may play regulatory roles in the responses to various abiotic stresses by controlling sugar allocation. For example, the *atsweet11/atsweet12* double mutants showed enhanced sugar accumulation in response to cold treatment, which further enhanced freezing tolerance [18]. Transgenic plants overexpressing *AtSWEET4* accumulated more glucose and fructose than wild-type plants, and displayed increases in plant size and freezing tolerance [73]. In the present study, almost half (12/25) of *MtSWEET* genes exhibited extensive responses to cold, salt, and drought stresses (Figure 6; Table S2), and similar results were also observed in banana [6], tea plant [39], and cotton [59,74]. It is worth noting that the transcript levels of seven *MtSWEET* genes (*MtSWEET1a*, *MtSWEET2b*, *MtSWEET3c*, *MtSWEET9b*, *MtSWEET13*, *MtSWEET15c*, and *MtSWEET16*) were significantly altered by at least two of the three treatments (Figure 6 and Figure 7; Table S2). These results indicate that these genes may regulate stress responses by modulating sugar levels under stress conditions. Similarly, AtSWEET16 mediates the transport of fructose, glucose, and sucrose, and its expression decreased under cold stress. *AtSWEET16*-overexpressing plants exhibited remarkably increased freezing tolerance, probably due to an accumulation of sugars in the leaf under this stress [20]. Tea plant CsSWEET16 is also responsive to cold stress and functions in exporting fructose from vacuoles, which contributes to cold tolerance in transgenic *Arabidopsis* plants [39]. In addition, *MtSWEET1a* was suppressed under cold and drought stress conditions but induced by salt stress, while *MtSWEET13* was up-regulated by these treatments (Figure 6 and Figure 7; Table S2), implying that *M. truncatula* might have evolved different mechanisms to adapt to various abiotic stresses.

## 5. Conclusions

In summary, 25 *MtSWEET* genes were identified in *M. truncatula*, and their characteristics, chromosomal distributions, gene structures, and phylogenetic relationships were analyzed. In addition, the expression profiles of the *MtSWEET* genes in different developmental stages/tissues and in response to various abiotic stresses were also examined based on the microarray and RNA-seq data, respectively. Furthermore, the sugar transport abilities of MtSWEET proteins were determined by yeast complementation assays. Our findings provide important clues for further studying the biological functions of the MtSWEET proteins in the future.

## Figures and Tables

**Table 1:** *SWEET* gene family in *Medicago truncatula*.

Gene Name	Accession No.	Clade	Chromosomal Location	gDNA Size (bp)	CDS Size (bp)	Protein Physicochemical Characteristics			TMHs
						Length (aa)	MW (kDa)	pI	
*MtSWEET1a*	Medtr1g029380	I	chr1: 10054058..10056439 (+)	2382	735	244	26.95	9.36	7
*MtSWEET1b*	Medtr3g089125	I	chr3: 40831984..40834426 (−)	2443	744	247	27.50	9.36	7
*MtSWEET2a*	Medtr8g042490	I	chr8: 16377347..16378975 (+)	1629	708	235	26.11	8.70	7
*MtSWEET2b*	Medtr2g073190	I	chr2: 30983345..30991272 (−)	7928	2046	681	73.63	8.75	15
*MtSWEET3a*	Medtr3g090940	I	chr3: 41296423..41298378 (+)	1956	753	250	27.50	9.09	7
*MtSWEET3b*	Medtr3g090950	I	chr3: 41306407..41308335 (+)	1929	753	250	27.74	9.23	7
*MtSWEET3c*	Medtr1g028460	I	chr1: 9609945..9612620 (−)	2676	771	256	28.13	9.30	7
*MtSWEET4*	Medtr4g106990	II	chr4: 43976500..43978913 (−)	2414	717	238	27.32	9.58	6
*MtSWEET5a*	Medtr6g007610	II	chr6: 1673915..1676144 (−)	2230	723	240	27.02	5.88	7
*MtSWEET5b*	Medtr6g007637	II	chr6: 1699177..1703592 (−)	4416	747	248	27.52	9.06	7
*MtSWEET5c*	Medtr6g007623	II	chr6: 1684113..1685864 (−)	1752	702	233	26.22	8.96	7
*MtSWEET5d*	Medtr6g007633	II	chr6: 1694537..1696430 (−)	1894	708	235	26.41	9.08	7
*MtSWEET6*	Medtr3g080990	II	chr3: 36674946..36676295 (+)	1350	792	263	28.82	9.25	7
*MtSWEET7*	Medtr8g099730	II	chr8: 42223590..42226325 (−)	2736	771	256	27.94	9.05	7
*MtSWEET9a*	Medtr5g092600	III	chr5: 40443899..40445866 (−)	1968	759	252	28.43	9.15	7
*MtSWEET9b*	Medtr7g007490	III	chr7: 1515108..1517630 (+)	2523	762	253	28.59	8.18	7
*MtSWEET11*	Medtr3g098930	III	chr3: 45314680..45319675 (+)	4996	621	206	23.53	9.55	6
*MtSWEET12*	Medtr8g096320	III	chr8: 40403635..40405942 (+)	2308	768	255	28.52	9.80	7
*MtSWEET13*	Medtr3g098910	III	chr3: 45309861..45311769 (+)	1909	936	311	35.25	7.59	7
*MtSWEET14*	Medtr8g096310	III	chr8: 40382758..40385234 (+)	2477	837	278	31.40	8.59	7
*MtSWEET15a*	Medtr2g007890	III	chr2: 1184452..1186389 (−)	1938	867	288	32.63	6.43	7
*MtSWEET15b*	Medtr5g067530	III	chr5: 28543252..28545616 (+)	2365	810	269	30.40	7.61	7
*MtSWEET15c*	Medtr7g405730	III	chr7: 238597..240382 (+)	1786	807	268	30.05	8.88	7
*MtSWEET15d*	Medtr7g405710	III	chr7: 224711..226577 (+)	1867	840	279	31.43	9.09	7
*MtSWEET16*	Medtr2g436310	IV	chr2: 14123315..14129655 (+)	6341	741	246	27.02	9.10	7

## Supplementary Materials

The following are available online at https://www.mdpi.com/2223-7747/8/9/338/s1, Figure S1: Transmembrance helices of MtSWEET proteins. The distributions of TM helices were predicted using the TMHMM Server v. 2.0, Table S1: A list of primer sequences used in this study, Table S2: Differential expression analysis of *MtSWEET* genes involved in the responses to various abiotic stresses including cold, drought, and salt. The expression levels of the *MtSWEET* genes are shown as FPKM values.
