# Supplementary figures and images for: SWEET Gene Family in Medicago truncatula: Genome-Wide Identification, Expression and Substrate Specificity Analysis
Source: Plants (Basel). 2019 Sep 9;8(9):338. doi: 10.3390/plants8090338 (PMC6783836; doi:10.3390/plants8090338)

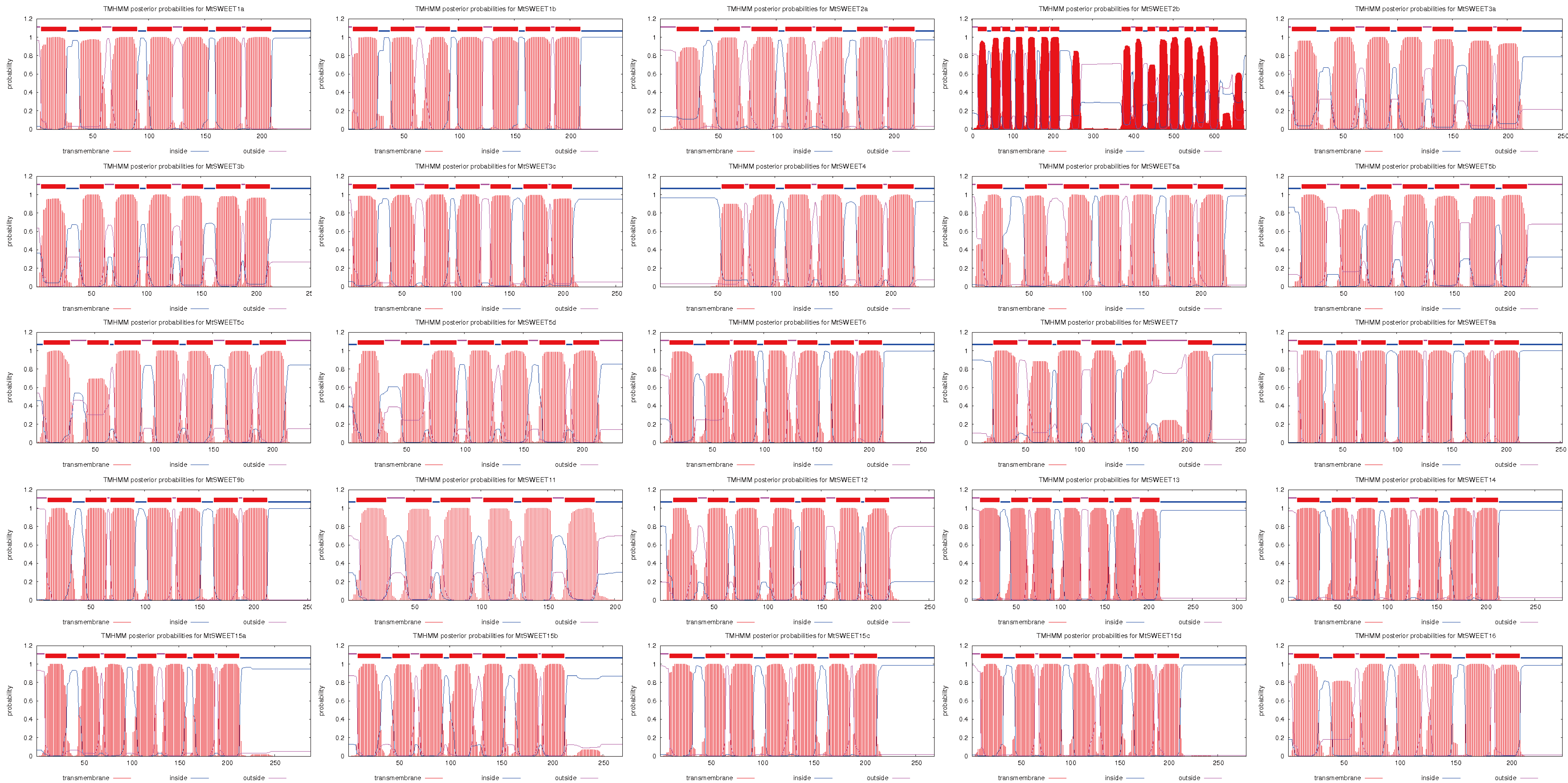

Supplement: Supplementary file 1 [file plants-08-00338-s001.zip › Fig. S1-R2.tif]
